# Toxins from Animal Venom—A Rich Source of Active Compounds with High Pharmacological Potential

**DOI:** 10.3390/toxins16120512

**Published:** 2024-11-27

**Authors:** Ekaterina N. Lyukmanova, Zakhar O. Shenkarev

**Affiliations:** 1 Faculty of Biology, Shenzhen MSU-BIT University, Shenzhen 518172, China; 2 Shemyakin-Ovchinnikov Institute of Bioorganic Chemistry, Russian Academy of Sciences, 117997 Moscow, Russia; 3 Moscow Center for Advanced Studies, 123592 Moscow, Russia; 4 Interdisciplinary Scientific and Educational School of Moscow University “Molecular Technologies of the Living Systems and Synthetic Biology”, Faculty of Biology, Lomonosov Moscow State University, 119234 Moscow, Russia

Animal venoms contain a huge variety of bioactive molecules—namely, toxins—with an almost combinatorial spectrum of biological activities. Since ancient times, venoms and individual toxins have attracted much attention, first as weapons against enemies and as active components in folk medicine, and nowadays as tools for functional and structural studies of essential membrane receptors and as prototypes in the design of new drugs [1]. Indeed, the diversity of venomous animals and the targets on which the components of their venoms act is extremely wide. Sea anemones, jellyfishes, cone snails, centipedes, spiders, scorpions, ants, bees, fish, frogs, lizards, snakes, and many other animals can produce venoms that are cocktails of highly toxic compounds. The spectrum of activity of these compounds is quite broad, and such activity is associated with the action on the basic functions of the prey which these poisonous creatures hunt or predators from which they defend themselves. These functions include breathing, mobility, cardiac activity, muscle contraction, nervous activity, pain signaling, blood coagulation, and integrity of tissues and individual cells, including blood cells.

Almost all these affected functions are related to the activity of different membrane receptors and ion channels. Moreover, given the combinatorial diversity of the venom components, it is possible to find an individual toxin acting on almost any given human receptor or ion channel, although in the most cases humans are not among the potential ‘preys’ or ‘eaters’ of these poisonous creatures. Thus, in venoms we can find toxins with activity on cationic Na^+^, K^+^, and Ca^2+^ channels, including voltage-gated [2], nucleotide-gated [3], and Ca^2+^-regulated channels [4]; chemo-, mechano-, and temperature-sensitive TRP channels [5,6]; anion Cl^−^ channels [7]; various ligand-gated channels including NMDA-R [8], GABA_A_-R [9], ASICs [10,11], and P2X receptors [12], as well as nicotinic acetylcholine receptors (nAChRs) [13,14], among others. Moreover, venoms contain toxins acting on various metabotropic receptors from the GPCR family, such as muscarinic acetylcholine receptors [15], as well as toxins acting on acetylcholinesterase [15], platelets [16] and cell membranes, causing their lysis (such as snake venom cytotoxins and phospholipases [17,18]) and disrupting the integrity of tissues (such as snake venom metalloproteinases, which damage the basement membrane of blood vessels, causing hemorrhage [17,18]).

It is not an exaggeration to say that the majority of targets of the toxins are pharmacologically relevant membrane receptors and ion channels in which researchers and pharmacological companies have a strong interest for drug design. There are already examples of drugs derived from animal toxins which have passed clinical trials and are presently used in medicine. These include drugs developed for the treatment of type 2 diabetes (Exenatide/Byetta^®^ and Lixisenatide/LyxumiaTM from Amylin and El Lilly, Sanofi), pain (Zinconitide/Prialt^®^ from Azur Pharma and Eisai), hypertension (Captopril/Capoten^®^ from Bristol-Myers Squibb; Enalapril/Vaotec^®^ from Bausch Health), and for perioperative bleeding (Batroxobin from Nuokang Biopharma), or drugs used as anticoagulant (Bivalirudin/Angiomax^®^ from The Medicine Co.) and antiplatelet agents (Eptifibatide/Integrilin^®^ from Merck; Tirofiban/Aggrastat^®^ from Iroko Cardio and Merck) [19]. Recently, the effective use of funnel web spider toxin Hi1a acting on the ASIC1a channel to reduce cardiac damage from heart attacks and to extend the life of transplanted heart was reported [20].

Great progress has been achieved in toxinology, in part due to the development of structural biology. Indeed, the determination of the spatial structures of toxin molecules, which is routinely performed by solution NMR spectroscopy [6,13], together with the determination of the spatial structures of membrane receptors and ion channels by cryo-electron microscopy (cryo-EM) [2], provide a solid basis for the rational design of targeted and specific drugs. Sometimes, structure–function studies of natural toxins and their targets point to the possibility for the creation of new types of therapy for previously untreatable diseases. For example, the observation that the crab spider toxin Hm-3 blocks leakage ω-currents through mutated variants of the human muscle-type Nav1.4 channel and identification of the mechanism of this interaction by NMR [21] point to the fundamental possibility of treatment of hyperkalemic or normokalemic periodic paralysis with appropriate ligands. The increased role of Artificial Intelligence (AI) systems in these structural studies is also worth mentioning. The success of AlphaFold-II [22] and other programs in prediction of the individual structures of membrane proteins [23] and toxins [24] is truly impressive. For example, the predicted structure of the Brazilian wandering spider toxin Pha1b corresponds very well to the experimentally determined NMR structure [6]. However, to date, progress in the prediction of structures of receptor–toxin complexes is less impressive. We believe that supplementing of structural studies with biochemical and physiological data will revolutionize the field of toxinology and the rational design of venom-based drugs in the near future.

The majority of the articles in this Special Issue, “Ion Channels, Venom, and Toxins”, describe new types of biological activities of recently discovered toxins acting on different ion channels. The published works not only illustrate the diversity of venomous organisms (sea anemones [10,13,14], spiders [6], scorpions [7,25,26], and snakes [11,18,27]) and the molecular targets of these toxins, but they also provide a snapshot of the variety of the structures used by nature to create these toxic molecules. These structures mainly include disulfide-stabilized peptide scaffolds such as the Inhibitor Cystine Knot (ICK) in the spider toxin Pha1b [6], sea anemone toxins Ms11a-1–Ms11a-4 [13], and short scorpion toxins Lqh 2-2, Lqh 7-1, and Lqh 8-6 [7]; the β-defensin APETx-like fold in the sea anemone peptides Hmg 1b-2, Hmg 1b-4, and Hmg 1b-5 [10,14]; and the three-finger Ly6/uPAR scaffold of the snake toxin mambalgin-2 [11]. The conservation of the ICK fold in evolutionarily distant organisms is not surprising. Peptides containing the ICK fold have been found in many eukaryotic phyla, including plants [28]. Two other types of structural folds presented in this Special Issue are also very ancient: for example, defensin-like molecules have been described in plants, animals and fungi, but probably have two independent evolutionary origins [29], while three-finger proteins have been found in many animal phyla, starting with Echinodermata [30]. The toxic molecules found in nature are also can be presented by larger proteins, such as viper phospholipases and metalloproteinases [18], and small organic compounds such as derivatives of natural 1,4-naphthoquinones (1,4-NQs) [31]. The proposed biomedical applications and mechanisms of action of the described toxins are very broad: from anxiolytic, analgesic, and anti-inflammatory activities connected with targeting of ASICs, TRPA1, and P2X7 ion channels [6,10,31] to anticancer activity mediated by inhibition of the α-ENaC/ASIC1a/γ-ENaC heterotrimeric channel [11] and regulation of the cholinergic system [13]. In addition to functional and structural studies, usage of toxins as new tools for study of ion channels in living cells was proposed [25]. Two reviews on snake and scorpion neurotoxins targeting the ion channels round out the Special Issue [26,27].

Despite the consideration of venoms as a potential ‘treasure chest of bioactive compounds’, at present, only a small part of toxins and venoms have been characterized pharmacologically. Research devoted to the search for new venom components targeting specific ion channels and other membrane receptors is actively continuing and is far from completion. Clearly, many questions regarding the usage of venom toxins as drugs remain open, such as questions surrounding their specificity, systemic toxicity, side effects, targeted delivery and whether they can be used by themselves or if the design of small molecules mimicking the toxins’ activity is required. We hope that the answers to these questions will be found in the near future.
